# Editorial: Possible Mechanisms to Explain Abdominal Fat Loss Effect of Exercise Training Other Than Fatty Acid Oxidation

**DOI:** 10.3389/fphys.2021.789463

**Published:** 2021-11-15

**Authors:** Chia-Hua Kuo, M. Brennan Harris, Jørgen Jensen, Ahmad Alkhatib, John L. Ivy

**Affiliations:** ^1^Laboratory of Exercise Biochemistry, College of Kinesiology, University of Taipei, Taipei, Taiwan; ^2^Department of Health Sciences, College of William and Mary, Williamsburg, VA, United States; ^3^Department of Physical Performance, Norwegian School of Sport Sciences, Oslo, Norway; ^4^Laboratory of Exercise Biochemistry, University of Taipei, Taipei, Taiwan; ^5^School of Health and Life Sciences, Teesside University, Middlesbrough, United Kingdom; ^6^Exercise Physiology and Metabolism Laboratory, Department of Kinesiology and Health Education, University of Texas at Austin, Austin, TX, United States

**Keywords:** high intensity exercise, adipose tissue metabolism, nutrient redistribution, weight loss, skeletal muscle adaptation, metabolic disease

Obesity is a major health problem throughout the world, being one of the leading risk factors for type 2 diabetes mellitus, high blood pressure, cardiovascular disease, and premature death (Després and Lemieux, 2006; Ritchie and Connell, 2007). According to the World Health Organization, obesity has tripled over the last 40 years. Thus, recommended methods for weight management have flourished during this time. The fundamental cause for weight gain is an energy imbalance between calories consumed and calories expended. Undeniably, there are a number of factors that can affect caloric expenditure and consumption. However, with strict caloric restriction body mass can be significantly reduced.

Restricting caloric intake alone, however, can result in as much lean mass loss as fat mass loss (Chaston et al., 2007; Weinheimer et al., 2010). Therefore, individuals who are overweight or obese should focus on reducing body fat rather than body mass. In fact, being over fat even when not overweight has greater prevalence and is more strongly related to disease risk than being overweight and lean (Maffetone and Laursen, 2020). Research has shown that combining caloric restriction with an appropriate exercise program can reduce the fat composition of the body while limiting or even preventing the loss of lean mass (Chomentowski et al., 2009; Weinheimer et al., 2010). However, the type of exercise, which is most effective for enhancing fat loss has come into question (Kuo and Harris, 2016).

The removal of body fat by its conversion to carbon dioxide or “fat burning” is the process most described to explain the fat reducing outcome of exercise training. Therefore, it has been hypothesized that the exercise program that maximizes the rate of fatty acid oxidation will result in the greatest reduction in fat mass over time. In general, fatty acid oxidation is maximized at an exercise intensity between 50 and 70% of maximum oxygen consumption (Jeukendrup and Wallis, 2005). With increasing exercise intensity above this threshold level, the rate of fat oxidation declines as the reliance on carbohydrates increases. As a result, low to moderate intensity aerobic training is commonly recommended for weight management programs. Recent research, however, does not support this recommendation. It has been clearly demonstrated that anaerobic high-intensity intermittent training, which relies predominately on carbohydrates as a fuel source, produces greater body fat reduction than continuous aerobic exercise even when the caloric expenditure is held constant for both modes of exercise (Tremblay et al., 1994; Trapp et al., 2008). Moreover, training under hypoxic conditions, which should limit fatty acid oxidation when compared with normoxic conditions, has been observed to result in a greater reduction in fat mass and greater increase in lean body mass (Chia et al., 2013).

In an insightful review article by Kuo and Harris (2016), they proposed that creating a negative energy balance in fat cells due to competition with skeletal muscle and other body tissues for circulating carbon sources may better explain the fat reducing outcome of exercise than the fat-burning model. In the following series of articles this concept is discussed in depth, as well as possible metabolic and hormonal alterations commensurate with high-intensity exercise training, which could facilitate a competition between fat cells and skeletal muscle for postprandial carbon and nitrogen.

In the opening article, Harris and Kuo present the concept that the primary mechanism underlying the intensity-dependent fat loss effect of exercise is due to a postprandial carbon and nitrogen redistribution to exercise stressed tissues, as opposed to adipose tissue, for fuel replenishment, tissue repair, and adapted responses. They further discuss the possible effects of meal and supplement timing when the nutrient demands of muscle are high to decrease abdominal fat accumulation while facilitating muscle recovery and development.

The relationship between obesity, specifically abdominal obesity, and type 2 diabetes and cardiovascular disease is presented by Kolnes et al. They continue their discussion by comparing the effects of different types of exercise training on energy expenditure and substrate utilization, and the impact these training methods have on adipose tissue function and body composition. This theme is continued by Delgado-Floody et al. in which they present new findings related to the effects of a 16-week high-intensity exercise training program on body composition, blood pressure, cardiorespiratory fitness, and substrate utilization during exercise among overweight prehypertensive and hypertensive patients. Additionally, Puengsuwan et al. report on the effects of a muscle stretching exercise program on overweight and obese middle-age and older adults. They observed a significant reduction in waist circumference and increase in percent fat free mass in their cohort, who trained 5 days per week for 15 weeks. Since muscle stretching is unlikely associated with increased fatty acid oxidation, they propose that this reduction in waist circumference after muscle stretching training was due to a redistribution of carbons from the abdominal region to challenged skeletal muscle.

Hormonal regulation of lipolysis during exercise is reviewed by Laurens et al. They discuss the effects of acute and chronic exercise on abdominal white adipose tissue lipolysis in lean and obese individuals. Of particular interest is the discussion on the regulation of lipolysis by catecholamines, atrial natriuretic peptide, and insulin. Also captivating, is their discussion on the effect contracting skeletal muscle has on adipocyte lipolysis via secretion of myokines such as the newly discovered growth and differentiation factor 15 (GDF15). Growth hormone is also a vital lipolytic hormone with strong influence on central abdominal fat stores (Rudman et al., 1990). Reductions in visceral adipose tissue and insulin resistance have been demonstrated in obese adults with growth hormone therapy (Johannsson et al., 1997; Nam et al., 2001). In the review by Sabag et al., factors contributing to exercise-induced growth hormone response, and how this response influences visceral adipose tissue and cardiometabolic health is evaluated.

With adipocyte hypertrophy, there are substantial changes in cell function, enzyme activity, and the secretion of various adipokines. A reduction in omentin is associated with an increase in metabolic risk factors (De Souza Batista et al., 2007), while an increase in vaspin appears to be an intrinsic compensatory response to insulin resistance, atherosclerosis, and chronic inflammation (Youn et al., 2008; Kobat et al., 2012). The effects of high-intensity interval training on body composition, inflammatory markers, and the adipokines vaspin and omentin in diet-induced obese rats are presented by Costa et al. Another important adipokine is irisin, which possesses anti-obesity and anti-diabetic properties (Boström et al., 2012; Rodríguez et al., 2017). De Oliveira et al. discuss their findings related to the independent and combined effects of diet and exercise on irisin and visceral adiposity. Exercise has also been found to affect carbon distribution by altering the activity of hormone sensitive lipase (HSL). Liu et al. present their findings on the response of HSL to acute and chronic high-intensity intermediate training and moderate-intensity continuous training in adipose tissue of mice.

Diet is also explored in this series of articles. Research indicates that in comparison with low-fat diets, low-carbohydrate or ketogenic diets result in greater weight reduction and a better metabolic profile (Sharman et al., 2004; Volek et al., 2004). On this subject, Kong et al. report on the surprising beneficial effects of a 4-week ketogenic diet on body composition and cardiorespiratory fitness in overweight and obese Chinese females.

Finally, the effect of genotype on exercise-induced weight loss is presented by Cardoso et al. Specifically, they report on the influence of the Pro12Ala polymorphism of the PPARγ2 gene on the body composition of previously inactive participants in response to a 12-week aerobic exercise training program.

In summary, this Research Topic addresses the means by which exercise training reduces fat mass. Specifically, it offers support for the hypothesis that exercise training results in the redistribution of postprandial carbon and nitrogen from adipose tissue to skeletal muscle and other exercise-stressed tissues of the body, and provides several mechanisms by which this could occur. However, much is still unanswered regarding the means by which high-intensity exercise results in fat loss and should be the focus of future research. For example, more information is needed regarding the relative contribution of fat loss from fat cell death and adipocyte fatty acid oxidation after high-intensity exercise. It would be advantageous to determine the optimal hormonal profile associated with fat loss and the most appropriate exercise and diet program required to achieve such a hormonal profile. It would also be of benefit to identify peptides and nucleotides released from exercising muscle that could potentially induce myogenic differentiation of circulating stem cells, and the impact this could have on exercise-stressed tissue regeneration and development, as well as fat cell metabolism. Such information is ultimately important for developing the most appropriate and efficient exercise programs for reductioning fat mass and treating the numerous metabolic disorders and physical disabilities associated with obesity.

## Author Contributions

JI wrote the initial draft of the editorial. All authors reviewed and edited the initial draft and agreed with the final version of the editorial.

## Conflict of Interest

The authors declare that the research was conducted in the absence of any commercial or financial relationships that could be construed as a potential conflict of interest.

## Publisher's Note

All claims expressed in this article are solely those of the authors and do not necessarily represent those of their affiliated organizations, or those of the publisher, the editors and the reviewers. Any product that may be evaluated in this article, or claim that may be made by its manufacturer, is not guaranteed or endorsed by the publisher.
